# Highly sensitive low field Lorentz-force MEMS magnetometer

**DOI:** 10.1038/s41598-021-01171-z

**Published:** 2021-11-04

**Authors:** Sofiane Ben Mbarek, Nouha Alcheikh, Hassen M. Ouakad, Mohammad I. Younis

**Affiliations:** 1 Physical Science and Engineering Division, King Abdullah University of Science and Technology, Thuwal, 23955-6900 Saudi Arabia; 2 Mechanical and Industrial Engineering Department, College of Engineering, Sultan Qaboos University, Al-Khoudh, 123, PO-Box 33, Muscat, Oman

**Keywords:** Mechanical engineering, Characterization and analytical techniques

## Abstract

We present a highly sensitive Lorentz-force magnetic micro-sensor capable of measuring low field values. The magnetometer consists of a silicon micro-beam sandwiched between two electrodes to electrostatically induce in-plane vibration and to detect the output current. The method is based on measuring the resonance frequency of the micro-beam around the buckling zone to sense out-of-plane magnetic fields. When biased with a current of 0.91 mA (around buckling), the device has a measured sensitivity of 11.6 T^−1^, which is five orders of magnitude larger than the state-of-the-art. The measured minimum detectable magnetic field and the estimated resolution of the proposed magnetic sensor are 100 µT and 13.6 µT.Hz^−1/2^, respectively. An analytical model is developed based on the Euler–Bernoulli beam theory and the Galerkin discretization to understand and verify the micro-sensor performance. Good agreement is shown between analytical results and experimental data. Furthermore, the presented magnetometer is promising for measuring very weak biomagnetic fields.

## Introduction

In recent years, magnetometers have been extensively used in a wide range of applications, including biomedical, automotive industry, robotics, and nondestructive material testing^1–4^. In addition, the significant improvement in the performance of magnetic sensors, such as excellent sensitivity, high resolution, minimum detectable magnetic field, low power consumption, high stability, wide bandwidth, small size, low cost, and excellent linearity, has allowed them to be used in navigation (1 nT–600 μT), biomagnetic (100 fT–0.1 μT), and archeology (1 pT–700 µT) applications^5^. Some applications, such as inertial navigation system require high sensitivity and low noise^6^, whereas small size sensors are well-suited for probing microscopy^7^. In scanning Hall probe microscopy, miniature magnetic sensors can be integrated with temperature sensors for temperature compensation, thereby reaching a better trade-off between magnetic performance and spatial resolution^1,7^.

Various types of magnetic sensors have been developed, such as magnetoresistive and Hall-effect magnetometers, which have attracted attention due to their high sensitivity and reliability^8,9^. However, these magnetometers suffer many challenges, such as hysteresis effect and incompatibility of the material with standard manufacturing processes^10^. Micro-electromechanical Systems (MEMS) magnetometers with low power consumption and low cost have emerged as a promising alternative^11–13^. In addition, these magnetometers can be implemented on silicon without the need for any special magnetic materials^13^. Recently, considerable efforts have been focused on resonant Lorentz-force MEMS magnetometers^14–19^, which are based on measuring the resonance frequency of a microstructure using optical^20,21^, piezoresistive^17,22^, and capacitive techniques^23,24^.

The optical sensing technique needs a simple read-out circuitry and has good immunity to electromagnetic interference^23^. However, this method is bulky and cannot be easily integrated with the sensor for mass production. The piezoresistive method is a simple sensing technique that requires an easy fabrication process^23^. Nevertheless, the technique can have voltage offset and temperature dependence issues^5^. The capacitive sensing technique is the most used owing to its low noise and low power consumption^25^. However, it suffers from parasitic capacitances. Still it has low temperature dependence^19^.

Efforts have been made to improve the sensor performance, such as its sensitivity and resolution, by using different approaches, which can be classified into extrinsic and intrinsic techniques. The extrinsic technique involves the new modulation and control systems used to drive the MEMS Lorentz force sensors, either using open- or closed-loop^12,26–30^. The closed-loop aims to operate the sensor exactly at mechanical resonance^26^. Using the closed-loop control improves significantly the dynamical behaviour of the MEMS sensors, which enhances the measurement accuracy^31^. However, this approach needs a lock-in amplifier that increases the circuit complexity and complicates the integration of the control system with the MEMS device^32^. Compared to the closed-loop, the open-loop approach is compact with less complexity for the driving and sensing electronics in spite of the sensitivity to parameter uncertainties of the input signal^32^. On the other hand, the intrinsic technique can be attributed to the device itself using advanced MEMS capabilities^23^. Many research studies have been reported to amplify the Lorentz force and thus boost sensitivity using internal amplification^17^ or micro-leverage mechanism^14^. Moreover, several MEMS dynamical phenomena have been utilized to improve the MEMS sensor sensitivity^19,33,34^, such as veering and buckling.

In a previous work, we proposed a highly sensitive Lorentz-force magnetic micro-sensor based on an in-plane electrothermally heated initially curved micro-beam exhibiting the veering phenomenon between its first two symmetric vibration modes^35^. In a recent work, we presented a miniature highly sensitive wide-range resonant magnetic Lorentz-force micro-sensor based on measuring the resonance frequency of straight heated micro-beam operating around the buckling zone^36^. The resonance frequency is measured with optical sensing (laser) and the sensor is operated at ambient pressure. In this paper, we explore the performance of a similar sensor to detect experimentally low magnetic field using open loop control system (capacitive sensing). We show that the proposed sensor is capable of detecting a magnetic field of 100 μT. In addition, a Galerkin-based reduced-order model (ROM) is developed to understand and verify the micro-sensor performance, which can further help in the designing of ultra-high sensitivity magnetometer.

### Micro-sensor working description and principle of operation

**Figure 1:**
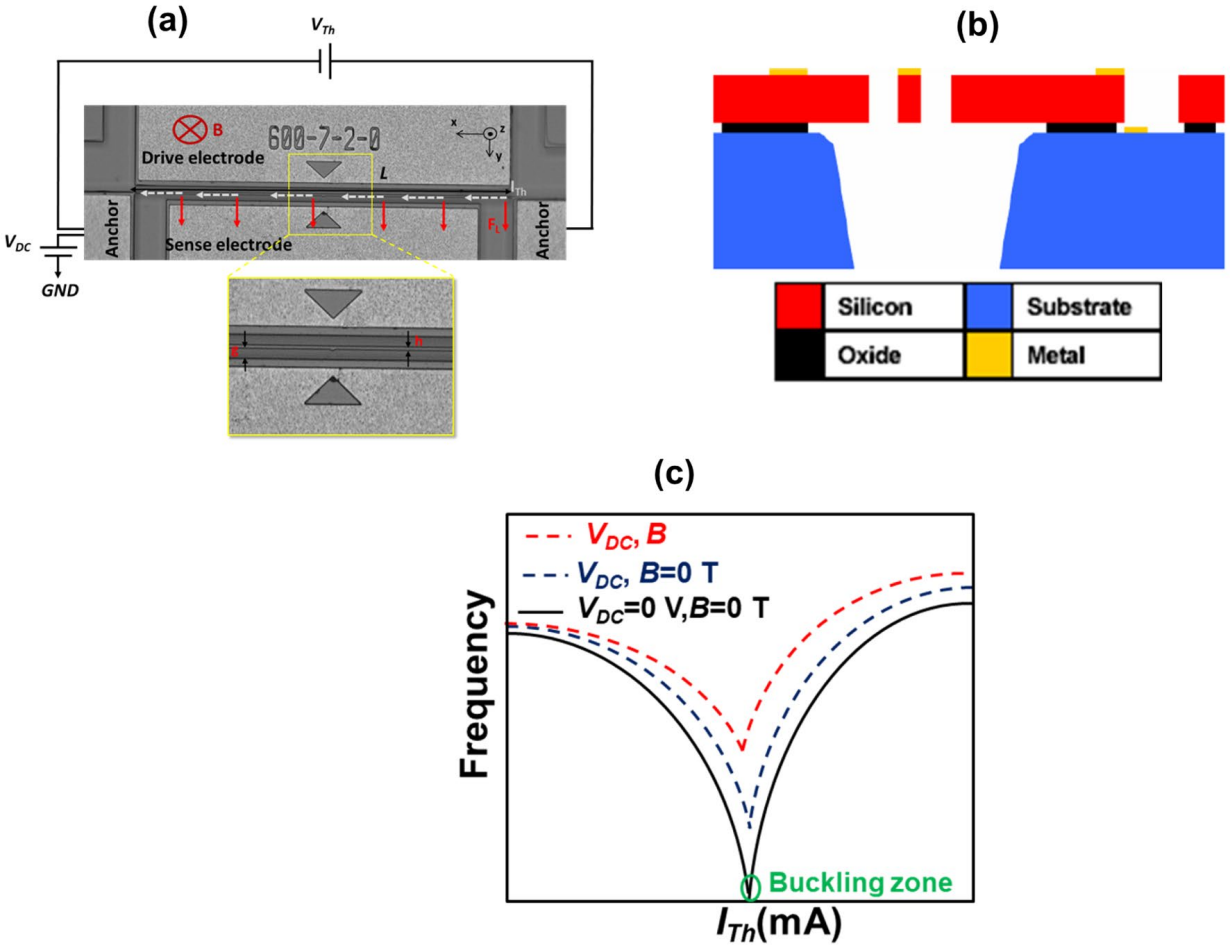
(**a**) Optical image of the in-plane clamped–clamped micro-beam with the geometrical parameters. (**b**) Cross-sectional view of the fabricated structure by SOIMUMPs. (**c**) Schematic illustrating the shift in the first resonance frequency of the micro-beam under input current (*I*_*Th*_), bias DC static voltage (*V*_*DC*_), and flux magnetic field (*B*).

Figure 1a shows an optical image of the micro-sensor. It consists of a clamped–clamped straight micro-beam fabricated from SOIMUMPs process by MEMSCAP^37^. The fabrication process is based on highly doping 30 µm Si device layer of Silicon On-Insulator SOI substrate. As seen in Fig. 1a, the micro-beam is sandwiched between two electrodes to electrostatically induce in-plane vibration and to detect the output AC current. Figure 1b shows a cross-sectional view of a SOIMUMPs processed device similar to the studied one. The micro-beam has a 600 μm in length (*L*), 2 μm in width (*h*), and 30 μm in thickness (*b*). The gap (*g*) between one of the electrodes and the resonating micro-beam is 7 μm. Applying an electrothermal voltage (*V*_*Th*_) between the anchors of the micro-beam, generates an induced current (*I*_*Th*_), through Joule’s heating, which causes a compressive axial stress^38^. Increasing *I*_*Th*_ leads to increase in the compressive axial load, and thus the resonance frequency (stiffness) of the micro-beam decreases. Increasing further *I*_*Th*_, the resonance frequency decreases until reaching a minimal value (very low stiffness). This is known as the buckling phenomenon^39^, see Fig. 1c. In addition, the micro-beam is electrostatically actuated with a static DC voltage to avoid the dip of the resonance frequency near buckling (bias the buckling), Fig. 1c.

**Figure 2:**
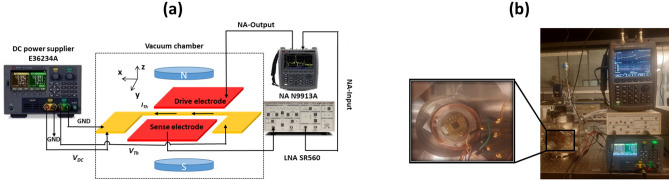
(**a**) Schematic of the experimental setup. (**b**) Photo of the experiment platform.

In this work, the proposed MEMS magnetometer is based on the Lorentz force principle. Hence, to generate a constant magnetic flux *B,* the device is placed inside two identical circular magnetic coils, which is measured using a Gaussmeter with a commercial Hall probe. A schematic of the experimental setup and a photo of the experiment platform are shown in Fig. 2a,b, respectively. As seen in the Fig. 2a, to provide an AC harmonic voltage (*V*_*AC*_), the drive electrode is connected to the network analyzer output (Keysight N9913A). To sense the output AC motional current (*I*_*O*_) due to the micro-beam in-plane motion, the sense electrode is connected to the input of the network analyzer. In addition, we use a low-noise amplifier (LNA SR560) to amplify *I*_*O*_. Note that the existence of *V*_*DC*_ creates an initial deflection, which enhances the output motional current. As we can see from Figs. 1a and 2a, with the presence of a magnetic field (*B*) in the -z-axis and with a DC current (*I*_*Th*_) in the + x-axis, Lorentz-force (*F*_*L*_) is generated normal to the micro-beam in the + y-axis. As shown in^21,36^, to achieve maximum sensitivity, the Lorentz force is chosen to be in the same direction of the initial deflection and buckling direction (+ y-axis).

## Dynamic analytical model

Assuming an Euler–Bernoulli nonlinear beam model, the structural in-plane behaviour of the micro-beam is governed by the following equation and boundary conditions:

1 $$\left\{\begin{array}{c}EI\frac{{\partial }^{4}w\left(x,t\right)}{\partial {x}^{4}}+\rho A\frac{{\partial }^{2}w\left(x,t\right)}{\partial {t}^{2}}+\tilde{c }\frac{\partial w\left(x,t\right)}{\partial t}=\left(\frac{EA}{2L}{\int }_{0}^{L}\left[{\left(\frac{\partial w}{\partial x}\right)}^{2}\right]dx-\frac{EA}{L}{\int }_{0}^{L}\alpha \left(T\left(x\right)-{T}_{amb}\right)dx\right)\frac{{\partial }^{2}w\left(x,t\right)}{\partial {x}^{2}}+\\ +\frac{{\varepsilon }_{0}b{V}_{DC}^{2}}{2{\left(g-w\left(x,t\right)\right)}^{2}}+B{I}_{Th}\\ w\left(x=0,t\right)=w\left(x=L,t\right)=0; \, \, \, \, \, \frac{\partial w\left(x=0,t\right)}{\partial x}=\frac{\partial w\left(x=L,t\right)}{\partial x}=0; \, \, \end{array}\right.;$$

where $w\left( {x,t} \right)$ denotes the in-plane (along *y*-direction) displacement in space *x* and time *t*, *ρ* is mass density, *E* is the Young’s modulus, *A* = *bh* and *I* = *bh*^3^/12 are the cross-sectional area and second moment of inertia, $\tilde{c}$ represents the overall viscous damping coefficient, *α* is the thermal expansion coefficient, ${T}_{amb}$ is the ambient temperature, and ${\varepsilon }_{0}$ is the dielectric constant of air. The space-dependent temperature profile $T\left( x \right)$ can be obtained through solving the steady-state heat transfer equation as detailed in^40^.

The resultant equations and boundary conditions system, Eq. (1), are numerically discretized using a Galerkin’s modal based expansion technique^41^. Accordingly, the micro-beam deflection is expanded as follows:

2 $$w\left(x,t\right)={\sum }_{i}{\psi }_{i}\left(x\right){u}_{i}\left(t\right); \, (1\le i\le N)$$

where $\psi_{i} \left( x \right)$ represents the mode-shape of an un-forced beam and $u_{i} \left( t \right)$ is the corresponding unknown time-dependent modal coordinate amplitude. Next, Eq. (2) is substituted into Eq. (1). After that, the resultant outcome is multiplied by the mode-shape functions $\psi_{j} \, (1 \le j \le N)$ and the subsequent *N*-equations are then integrated from 0–*L*. This produces *N* Ordinary-Differential Equations (ODEs) in $u_{j} \left( t \right) \, (1 \le k \le N)$ forming a Reduced-Order-Model (ROM). A convergence analysis was carried out and it was found that five modes (*N* = 5) is sufficient for convergence. The derived ROM can be used to calculate the micro-resonator natural frequencies. Toward this, the static deflection of the beam is first calculated. For this, we evaluate the stationary deflection by setting all time dependent terms in the ROM equal to zero. Then the modal amplitudes $u_{i} \left( t \right)$ are replaced by unknown constant coefficients *a*_*k*_. This leads to a system of nonlinear algebraic equations that can be solved numerically using Newton–Raphson algorithm. Finally, the eigenvalue problem of the micro-resonator is solved by perturbing the response around the calculated static position and considering only the linear part of the ROM. Further detail of the eigenvalue problem can be found in^42^.

## Results and discussion

**Figure 3:**
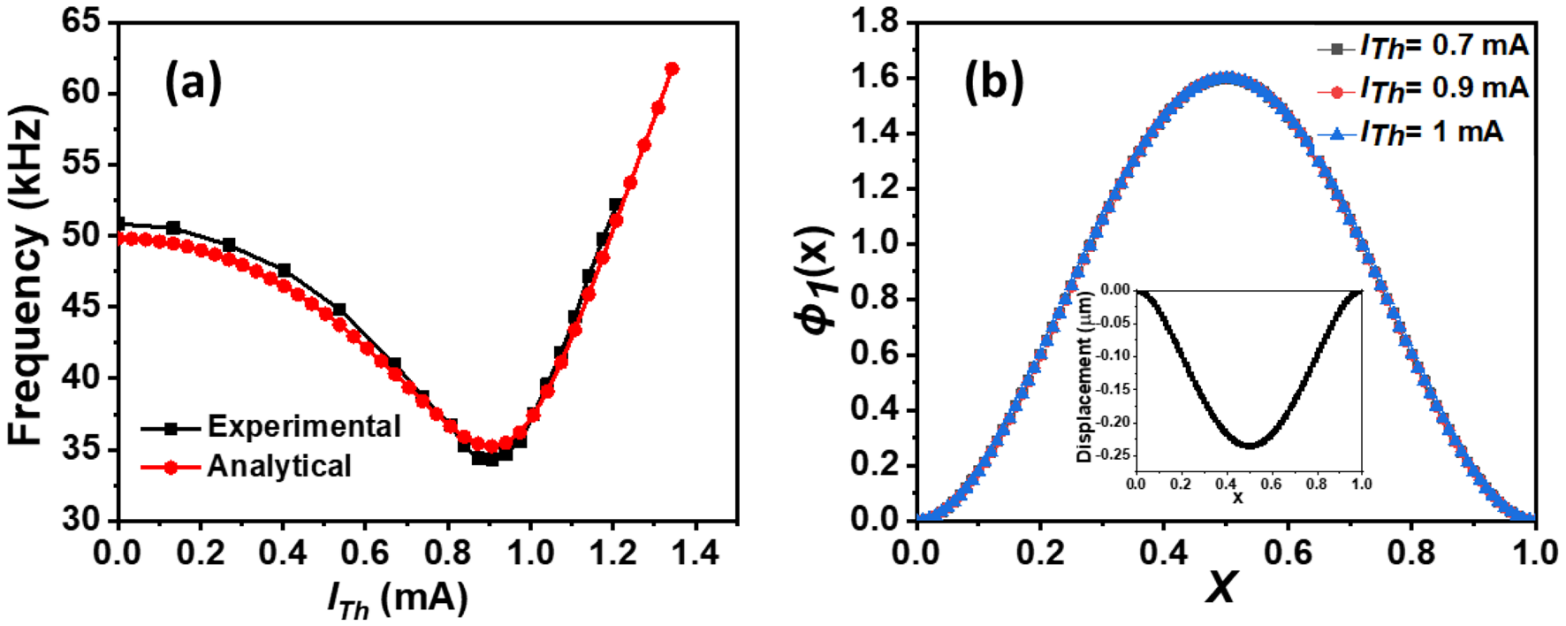
(**a**) Experimental and analytical results of the resonance frequency variation of the micro-beam with the electrothermal current *I*_*Th*_. (**b**) The simulated mode shape for *V*_*DC*_ = 30 V and for various of *I*_*Th*_. The inset shows the analytical result of the static deflection of the micro-beam with *V*_*DC*_ = 30 V and *I*_*Th*_ = 0 mA.

To perform the measurements, the magnetometer is placed inside a vacuum chamber with a pressure of 6 mT. The DC voltage *V*_*DC*_ and the electrostatic driving rms amplitude *V*_*AC*_ are set to 30 V and 9.988 mVrms (− 27 dBm). Figure 3a shows the experimental and analytical results of the variation of the fundamental resonance frequency of the micro-beam while changing the electrothermal current *I*_*Th*_. Good agreement is shown among the results. It can be seen that the resonance frequency decreases with the increase of *I*_*Th*_ until it reaches the buckling zone (*I*_*Th*_ = 0.91 mA). Within this range, the resonance frequency varies from 52 to 35 kHz, confirming that the presence of *V*_*DC*_ causes perturbation around the buckling zone (imperfect buckling). Thus, the system shows a midpoint displacement of 0.25 μm (initial curvature), see the inset of Fig. 1a. Hence, operating around this zone, the micro-sensor is very sensitive to any stiffness change (for instance from the magnetic distributed forces). After the imperfect buckling zone, the resonance frequency increases due to the induced curvature of the micro-beam.

To further investigate the dynamic response of the system with *I*_*Th*_, we plot the mode shapes of the micro-beam for *V*_*DC*_ = 30 V and for various *I*_*Th*_, Fig. 3b. It is clear that, before the buckling point, there is no effect of the compressive axial load on the mode shapes.

**Figure 4:**
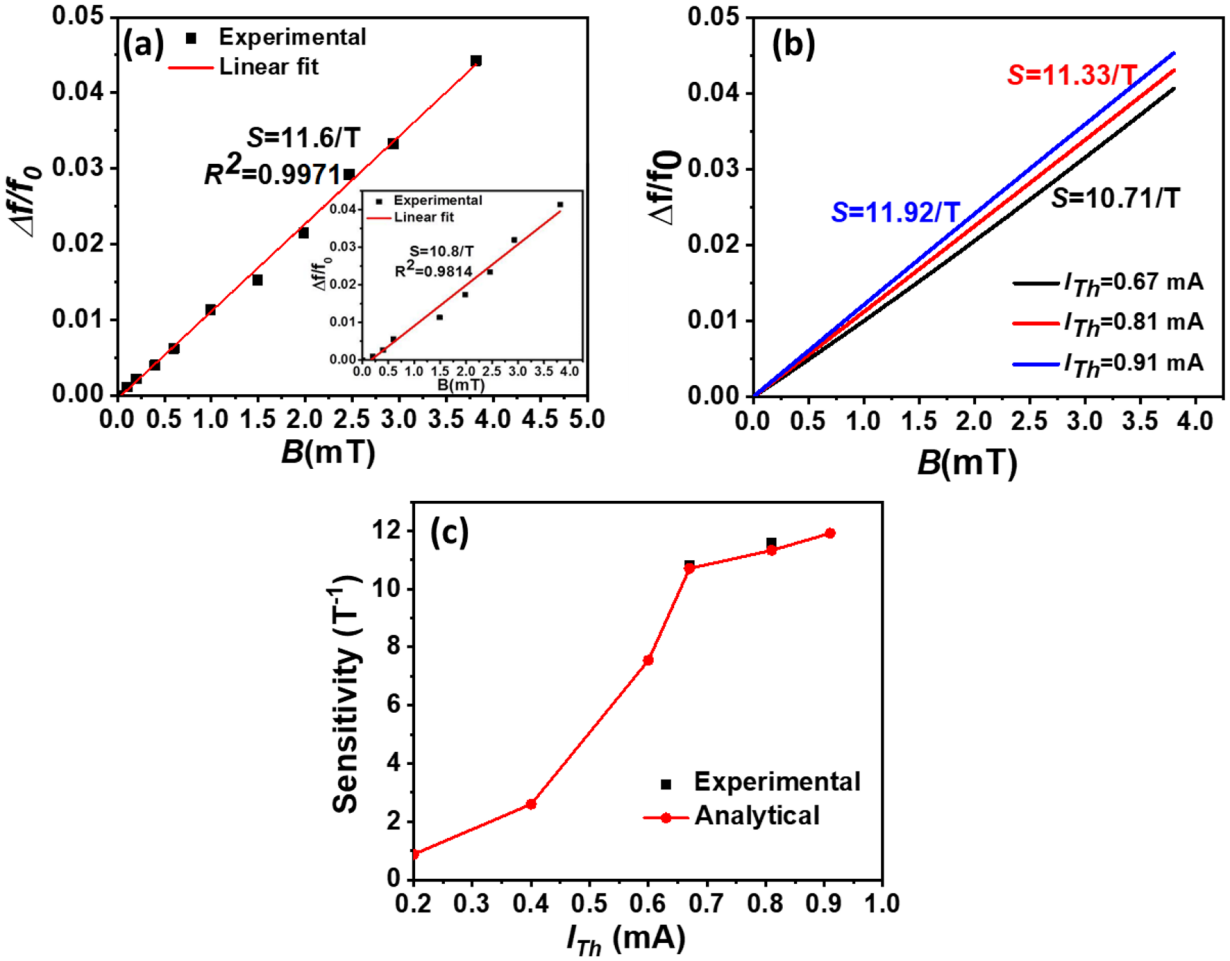
(**a**) Experimental results of the normalized resonance frequency variation versus input magnetic field for *I*_*Th*_ = 0.81 mA. The inset shows the normalized resonance frequency shift versus input magnetic field for *I*_*Th*_ = 0.67 mA. The slope of the plot presents the sensitivity of the device (*S*). (**b**) Analytical results of the variation of the normalized resonance frequency shift of the micro-beam for different *I*_*Th*_ values. (**c**) Experimental and analytical results of *S* versus *I*_*Th*_.

Here, the buckling bifurcation will be used to sense the out-of-plane magnetic field in the low ranges. The Lorentz-force alters the micro-beam stiffness, which increases its resonance frequency. This frequency shift forms the basis of the proposed micro-sensor. Figure 4a shows the variation of the normalized resonance frequency (*Δf/f*_*0*_) variation versus input magnetic field (*B*) for *I*_*Th*_ = 0.81 mA (around buckling point) where Δ*f* is the frequency shift, which is defined as (*f*–*f*_*0*_). *f* and *f*_*0*_ are the resonance frequency of the micro-beam with and without *B*. In Fig. 4a, the sensitivity can be evaluated as the slop of the variation *Δf/f*_*0*_ with *B*. As seen in Fig. 4a, the device capable of sense a wide range of *B* from 100 μT to 4 mT. Also, it can be seen that the sensor has high sensitivity (*S*) of 11.6 T^−1^. Further, we check the linearity of the proposed micro-sensor. Figure 4a demonstrates that the micro-sensor has high linearity less than 0.5% in the entire magnetic field range. Next, we plot the results for *I*_*Th*_ = 0.67 mA, inset of Fig. 4a. The result shows a measured *S* of 10.8/T with 2% of non-linearity. This value is lower than the value near the buckling point 0.81 mA. The analytical results of the variation *Δf/f*_*0*_ with *B* while changing *I*_*Th*_ are shown in Fig. 4b. The results agree with the experimental data, showing a high sensitivity of 11.92 T^−1^ at the buckling point (*I*_*Th*_ = 0.91 mA).

To further analyze the effect of the operating current on the sensitivity, Fig. 4c illustrates the analytical and experimental results of *S* with *I*_*Th*_ before the buckling zone. The theoretical results match well with the experimental data. As expected, *S* increases with the increase of *I*_*Th*_. At 0.91 mA, the sensor achieves a sensitivity that is 14 times higher than the sensitivity of 0.2 mA. The results confirm well the concept that by operating at the buckling point, the Lorentz-force magnetometer is extremely sensitive to out-of-plane magnetic fields. According to the results presented in Fig. 4b,c, the optimized sensitivity of the device can be achieved by carefully choosing the operating current *I*_*Th*_ to reach the bifurcation point.

Next, we discuss the total power consumption of the proposed magnetic sensor. It results from the combination of the electrostatic drive power of the resonator (in order of nW) and the electrothermal power^43^ (in order of mW). Thus, the overall power consumption is dominated by the electrothermal voltage. It is estimated based on resistive heating of the resonator (*V*_*Th*_^2^/*R*) where *R* is the micro-beam resistance. For 600 µm micro-beam, *R* is found to be 1.49 KΩ and with *I*_*Th*_ of 0.91 mA, the magnetometer power consumption (*P*) is around 1.2 mW. Moreover, *I*_*Th*_ can be decreased to decrease the power dissipation by Joule’s effect. This will be on the expense of decreasing the sensitivity, i.e., *S* ∝ *I*_*Th*_. It is important to note that there are many ways to improve the micro-sensor performance, including designing devices with low *R,* which reduces the dissipation and enhances the sensitivity.

Note here that the current detection method using capacitive sensing may suffer from parasitic capacitances and other sources of noise. Moreover, this sensing method can have an impact on the resolution of measurements. The measured quality factor *Q* of the sensor is found to be around 200. Thus, for the noise analysis of the system, we assume only the thermo-mechanical white noise (*Q* > 100 for frequency-modulated sensors) since the system shows a low noise voltage related to the LNA instrumentation amplifier around 4 nV.Hz^−1/2^^44^. Thus, the evaluated thermo-mechanical noise (Brownian limited resolution) of the proposed magnetic sensor can be expressed as^15,45^:

3 $$Noise=\frac{\sqrt{4{k}_{B}Tb}}{L{I}_{Th}}$$

where *k*_*B*_ is Boltzmann’s constant, *T* is absolute temperature in Kelvin, and *b* is the damping coefficient (*b* = 0.0025). The theoretical Brownian noise is found to be 13.6 µT.Hz^−1/2^ for a 0.81 mA input current. Clearly, a longer micro-beam can decrease the noise limit. The Brownian limited resolution may be decreased further by increasing the power or by lowering the damping of the system. Hence, the micro-sensor design can be adjusted to improve the noise performance.

**Figure 5:**
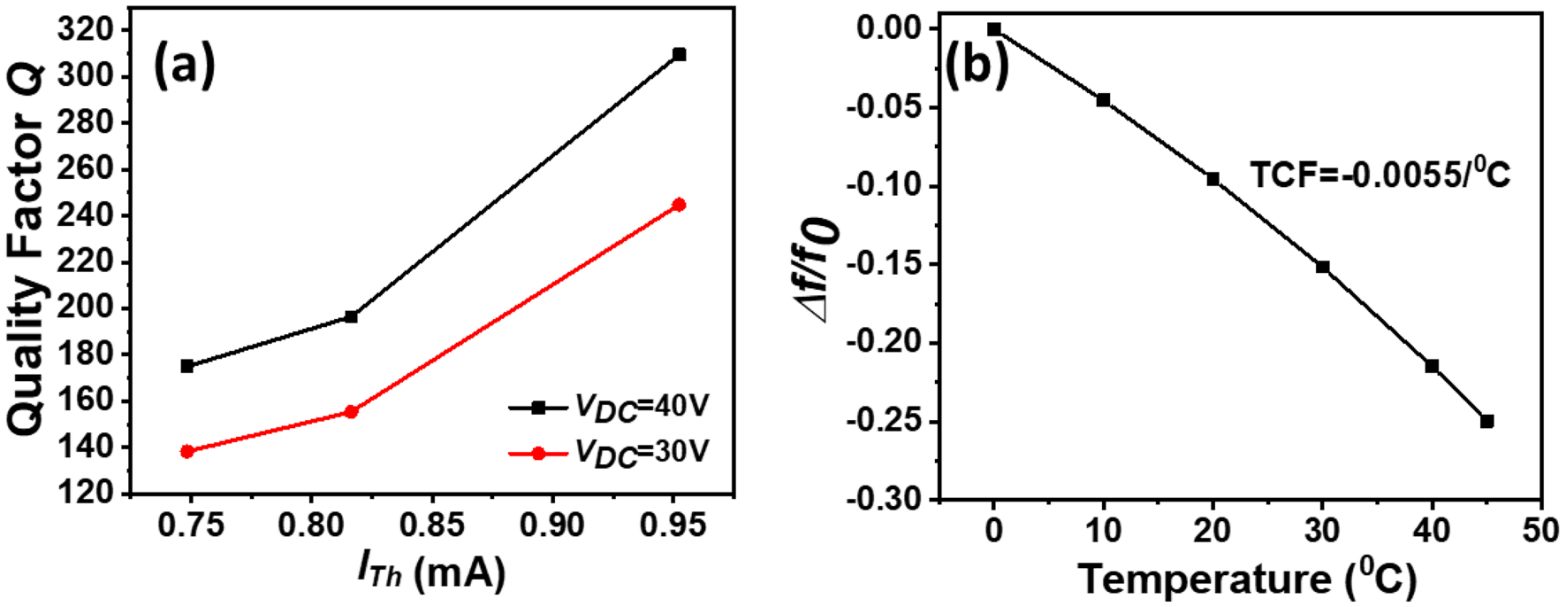
(**a**) Measured quality factor (*Q*) versus *I*_*Th*_ for *V*_*DC*_ = 30 V and 40 V. (**b**) Simulated results of the temperature dependence of frequency (TCF) of micro-resonator.

As we notice above, the thermo-mechanical noise is dependent on *Q*, and its magnitude can be affected by the input current. Hence, we measured the *Q* change versus *I*_*Th*_ for *V*_*DC*_ = 30 V and 40 V, see Fig. 5a. The results show an increase of *Q* as increasing *I*_*Th*_ or *V*_*DC*_. It is clearly noticeable that the sensor has the highest *Q* at the buckling zone. Thus, operating around the buckling zone, the proposed magnetometer exhibits high sensitivity, good linearity, and also high quality factor.

Also, high-performance magnetic sensors should have high sensitivity over a wide temperature range. To investigate the effect of the temperature on the sensor, we simulate the temperature dependence of the resonance frequency from 0 to 45 °C, see Fig. 5b. As can be seen in Fig. 5b, the results show a temperature coefficient of frequency (TCF) of − 0.0055/°C. For the temperature compensation, we can use a highly n-doped silicon when the temperature coefficient of the electrical stiffness becomes large enough to compensate or supersede the effect of the temperature coefficient of the mechanical stiffness^46^. However, at high doping, the increase of electrical stiffness may increase the operating point to higher bias currents.

**Figure 6:**
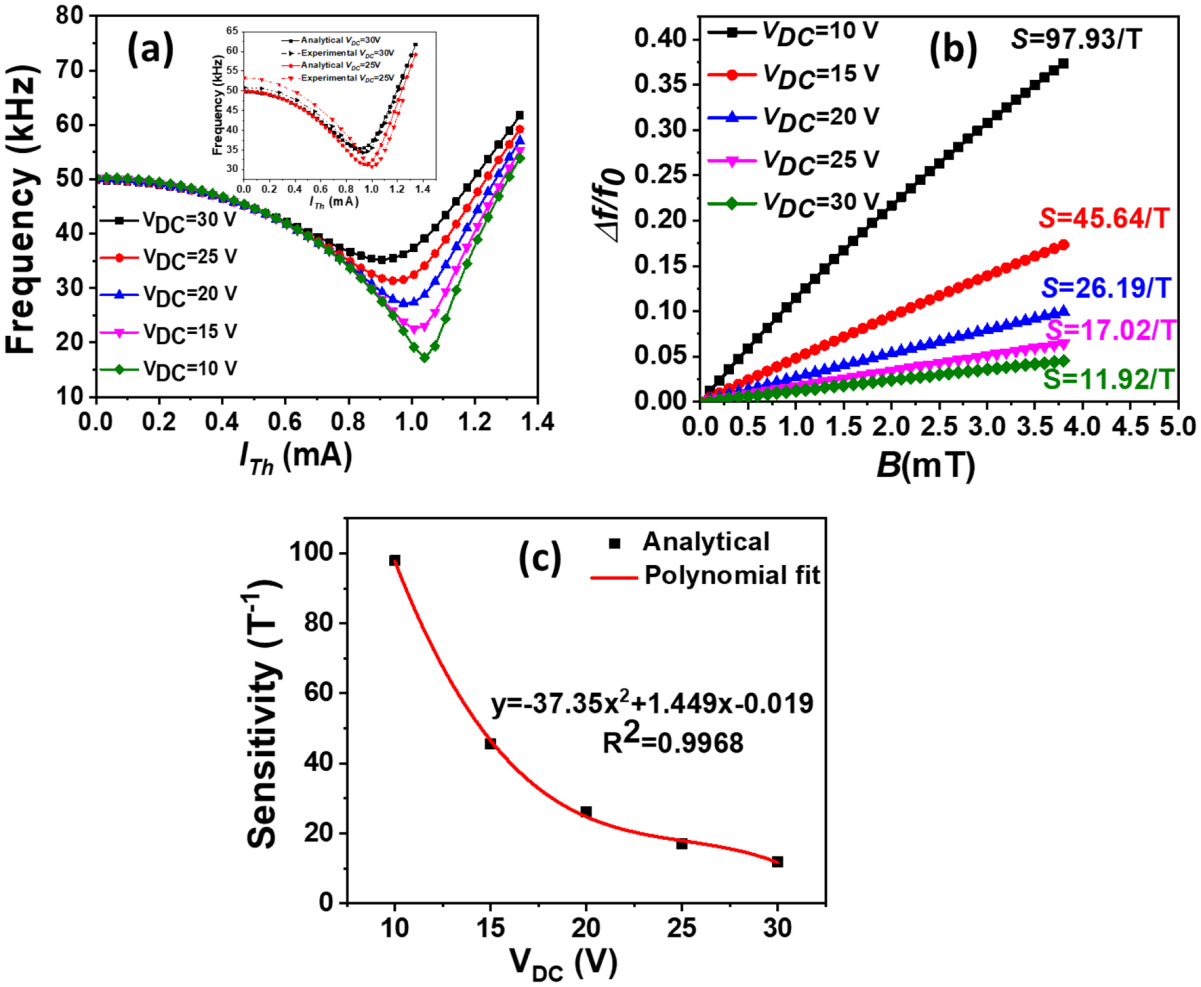
(**a**) Analytical results of the resonance frequency variation of the micro-beam with *I*_*Th*_ for various values of *V*_*DC*_. The inset shows the analytical and experimental variation of the resonance frequency for different *I*_*Th*_ for *V*_*DC*_ = 30 V and 25 V. (**b**) Results of the normalized resonance frequency variation versus *B* for different bias *V*_*DC*_. (**c**) Results of the variation of *S* versus *V*_*DC*_.

Further, analytical simulations are performed to predict the device performance. Figure 6 illustrates the effect of *V*_*DC*_ on the power consumption and sensitivity. Figure 6a shows the variation of the resonance frequency while varying *I*_*Th*_ for different values of *V*_*DC*_. The inset depicts a comparison between analytical and experimental results for *V*_*DC*_ = 30 and *V*_*DC*_ = 25 V.

As seen in the figure, as *V*_*DC*_ decreases, the corresponding resonance frequency drops until reaching a value of 17 kHz. In addition, its corresponding operating point increases to 1.05 mA. For low *V*_*DC*_ value (@ 10 V)_*,*_ the frequency response has the sharpest curve at the perturbed buckling zone (low stiffness), which can be extremely sensitive to any output forces. Figure 3b shows the results of *Δf/f*_*0*_ variation versus *B* for different bias *V*_*DC.*_

As can be seen in Fig. 6b, the increase in *Δf/f*_*0*_ follows a linear function with *B* for different values *V*_*DC*_*.* For further study of the effect of *V*_*DC*_ on the sensor sensitivity, we plot *S* as increasing *V*_*DC*_, Fig. 6c. The results show that as the actuation *V*_*DC*_ increases, the *S* decreases as a quadratic polynomial. Subsequently, first *S* drops rapidly, and then slowly decreases, as *V*_*DC*_ increases. Decreasing *V*_*DC*_ from 30 to 10 V, the sensitivity can be further improved by 920%. Indeed, these results demonstrate that the sensitivity is highly dependent on the applied *V*_*DC*_. In the proposed sensor with high airgap, adding high value of *V*_*DC*_ is necessary to improve the motional current on the sensing electrode. Accordingly, future efforts should minimize electrostatic driving *V*_*DC*_ with ultra-small air-gap size to make the output signal large enough to be sensed.

**Figure 7:**
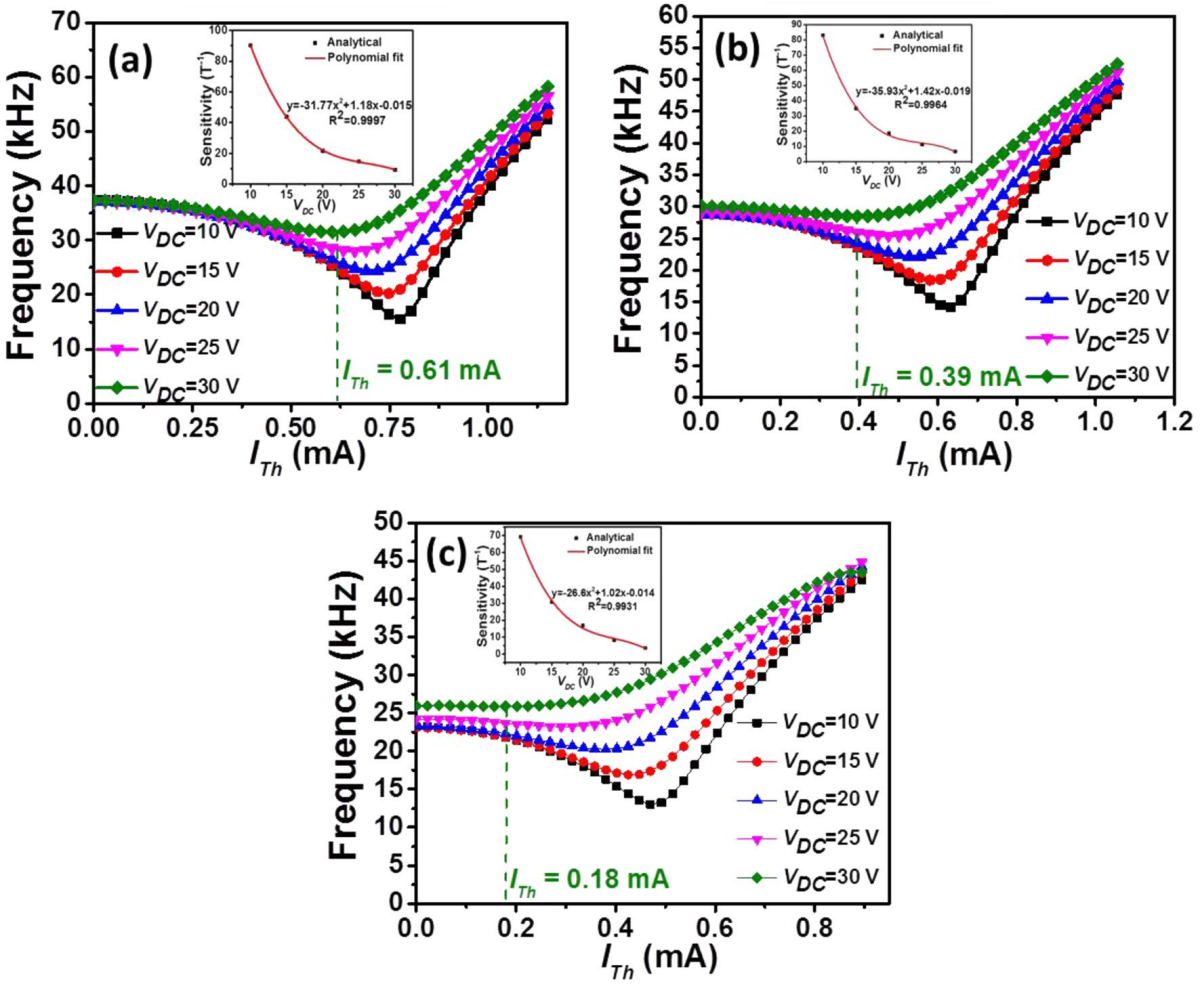
The simulated variation of the resonance frequency versus *I*_*Th*_ for various values of *V*_*DC*_ and different micro-beam’s length *L* (**a**) *L* = 700 μm, (**b**) *L* = 800 μm, and (**c**) *L* = 900 μm. The insets show the corresponding *S* versus voltage *V*_*DC*_.

**Figure 8:**
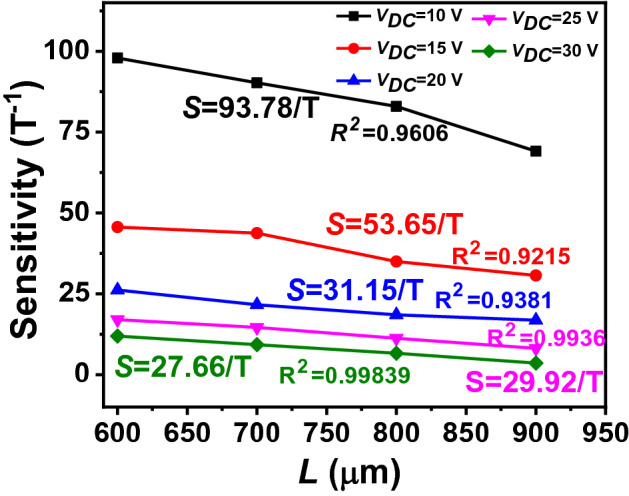
Sensitivity variation with *V*_*DC*_ for different micro-beam length *L*.

As we mention above, the device sensitivity is defined as the fractional change in frequency per unit of magnetic field density, which can be expressed as^14,15,30^^:^

4 $$S=\frac{\partial \left(\frac{\Delta f}{{f}_{0}}\right)}{\partial B}=\frac{{I}_{Th}L}{2k}$$

where *k* is the micro-beam’s stiffness. As can be seen from Eq. (4), *S* can be further enhanced by modifying the physical dimensions of the device more carefully or taking advantages from more complaint micro-beams. On the other hand, as implied by Eq. (4), increasing *L* or reducing* h* increases *S*. Hence, it responds more to the Lorentz-force for the same given magnetic flux. This explains that the device performance depends strongly on the geometrical parameter of the micro-beam. In the following, we investigate the influence of the micro-beam length on the resonance frequency and sensitivity. Based on the analytical model, we increase *L* from 700 μm to 900 μm and compute the resulting resonance frequencies. Figure 7 displays the results as tuning *I*_*Th*_ and *V*_*DC*_ for micro-beams with different lengths *L*. This figure shows that the initial natural frequency at *I*_*Th*_ = 0 mA decreases as *L* increases. In addition, we observe that at *I*_*Th*_ = 0 mA, the frequency responses increase with *V*_*DC*_ for micro-beam lengths 800 μm and 900 μm, respectively. This is due to the mid-plane stretching that is dominate over electrostatic force for a longer micro-beam and large capacitive gap. As noted from the figure, increasing *L* shifts the position of the buckling point from high to low values of *I*_*Th*_. This can be explained from the increase of the micro-beam’s resistance and the decrease of its stiffness. Hence, these results can be useful to reduce the energy consumption. Increasing the length to 900 μm can reduce the power consumption by 94% (*P* = 0.07 mW). Operating around the buckling point, Fig. 8 presents the analytical results of the variation of *S* as a function of *V*_*DC*_ and for different lengths *L*. As shown, increasing *L* results in the decrease of the sensor sensitivity. *S* decreases to the minimum of 2.4 T^−1^ for *L* of 900 μm and *V*_*DC*_ of 30 V. We conclude here that to improve the sensor sensitivity, the micro-beam length *L* and the input current *I*_*Th*_ should be increased (*S* ∝ *I*_*Th*_* L*). However, in both cases there is the cost of increased power dissipation by Joule’s effect. It also shows that the operating current of the magnetic sensor and *V*_*DC*_ can be tuned to achieve higher sensitivity.

These results can be used as guidelines to choose the geometric parameters to improve the sensor performance. The power consumption can be reduced by a factor of 2 if a 900 μm long beam is used. Moreover, the device sensitivity is expected to improve after reducing *V*_*DC*_. More analytical studies can be conducted to investigate the effects of the length-to-thickness (*L*/*h*) ratio on the energy dissipation and sensitivity. One should mention that the proposed sensor design is flexible and can be optimized to measure ultra-low biomagnetic fields’ applications.

Table. 1 shows the performance comparisons of the proposed micro-sensor with some of the reported Lorentz-force-based magnetometers. For a fair comparison, only MEMS magnetometers with frequency-modulation (FM) output, which the proposed sensor belongs to, have been included. It can be seen from Table 1 that the proposed miniature magnetic sensor has higher sensitivity compared with the state of the art. Moreover, the high sensitivity, low-power consumption, and good linearity, make the sensor promising for wide range of applications.

**Table 1:** Summary of the sensor’s performance of this work compared with some of the reported frequency modulation (FM) Lorentz-force-based magnetic micro-sensors.

References	Magnetic range (mT)	Current (mA)	Surface (mm^2^)	Sensitivity (ppm/mA.T)	Sensing method
[Zhang et al. ^30^]	≤ 100	10	0.48	33.9	Capacitive
[Sonmezoglu et al. ^18^]	[0–0.4]	3.3	1	7337	Capacitive
[Bahreyni et al. ^32^]	[2.5–25]	6	0.27	325.7	Capacitive
[Laghi et al. ^47^]	[− 5 to 5]	0.21	0.3088	28 × 10^5^	Capacitive
[Li et al. ^15^]	[0–0.4]	0.9	0.08	5200	Capacitive
[Li et al. ^14^]	[− 66 to 66]	4	0.96	6687	Capacitive
[Alcheikh et al. ^36^]	[4–8]	0.27@16 V	0.0144	125 × 10^6^	Optical
This paper	[0.1–4]	0.91 @ 25 V	0.0012	12.74 × 10^6^	Capacitive
This paper	[0.1–4]	1.03 @ 10 V	0.0012	95.07 × 10^6^	Capacitive

## Conclusions

In this paper, we proposed an out-of-plane Lorentz-force magnetic micro-sensor by exploiting the buckling point bifurcation of a straight micro-beam. The concept is based on the frequency shift measured at different magnetic field strengths. We showed that the sensitivity of the proposed magnetic sensor is very high. In addition, we demonstrated analytically an optimal sensor design whose sensitivity can be further enhanced to around 920%. Also, the proposed sensor shows good linearity. These features, combined with low-power consumption, simplicity of fabrication, low-cost, and scalability, make the sensor promising for variety of applications. We demonstrated experimentally the capability of the magnetometer to sense a low magnetic field around 100 μT using open loop control system. Future research work will include a closed loop control system to improve the noise performance. It was found that the performance of the proposed magnetometer depends strongly on the micro-beam length, operating point, and the electrostatic driving voltage. One should mention that the micro-sensor design is flexible and can be optimized to measure ultra-low biomagnetic fields’ applications.
